# Toward a Set of Criteria to Decide Which STIs to Screen for in PrEP Cohorts

**DOI:** 10.3389/fpubh.2019.00154

**Published:** 2019-06-12

**Authors:** Chris Kenyon

**Affiliations:** ^1^HIV/STI Unit, Institute of Tropical Medicine, Antwerp, Belgium; ^2^Division of Infectious Diseases and HIV Medicine, University of Cape Town, Cape Town, South Africa

**Keywords:** STI screening, antimicrobial resistance, gonorrhea, chlamydia, *M. genitalium*, MSM, PrEP, Delphi consensus

## Abstract

Contemporary HIV preexposure prophylaxis (PrEP) cohorts are characterized by high rates of partner change and as a result have high and fairly stable prevalences of *N. gonorrhoeae* and *C. trachomatis*. The available evidence suggests that intensive 3-monthly screening in this setting does not have a large effect on the prevalence of these infections but results in high antimicrobial exposures. Gonorrhea/chlamydia screening may thus be doing more harm than good. Compelling arguments can, however, be made to screen for HIV, hepatitis C, and syphilis in PrEP cohorts. In this perspective piece, we explore the logical basis for deciding which STIs to screen for in PrEP cohorts. We propose that a Delphi consensus methodology is used to derive, assess, and apply a broadly accepted set of criteria to evaluate which STIs to screen for in these cohorts. Finally, to illustrate the utility of the process, we derive and apply our own list of criteria as to which STIs to screen for. This process leads to a controversial conclusion, namely that stopping gonorrhea/chlamydia screening in a controlled and phased manner may offer net health benefits to PrEP cohorts.

## Introduction

Preexposure prophylaxis (PrEP) refers to the use of antiretroviral medications to prevent HIV-infection in persons at high risk of HIV acquisition (1). One of the largest target PrEP populations are men who have sex with men (MSM) with high rates of partner change (1–3). The participants in the PrEP study typically report a mean of between 9.3 and 18 partners per 3 months (1, 4, 5). The combination of high rates of partner change and infrequent condom usage (particularly for oral sex) result in high equilibrium prevalence of a range of STIs including *Neisseria gonorrhoeae, Chlamydia trachomatis*, and *Mycoplasma genitalium*. As a result, PrEP guidelines commonly recommend frequent (3-monthly) and 3-site (anorectal, pharyngeal, and urethral) screening for gonorrhea/chlamydia—termed 3 ×3 screening (6–9). A recent systematic review found that intense screening was not associated with a decline in the prevalence of these infections—regardless of whether the screening was conducted every 3, 6, or 12 months (7). The high prevalence of *N. gonorrhoeae* and *C. trachomatis* in PrEP cohorts, however, means that screening for these infections (and treating all positives which is standard practice) exposes around 20% of PrEP recipients to antimicrobials every 3 months (10). This results in macrolide exposures of up to 4,400 standard doses/1,000 population per year (10). This exposure level is 41 times greater than that of populations such Latvia and considerably higher than exposure levels found to be strongly associated with the induction of antimicrobial resistance (AMR) in a range of bacteria including *N. gonorrhea* and *Treponema pallidum* (10–15).

A range of different studies have revealed that a key way to prevent the emergence of AMR is reducing the consumption of antimicrobials to below resistance inducing thresholds (14, 16). Populations that have kept antimicrobial consumption below such thresholds tend to have low rates of AMR for a range of bug-drug combinations whereas populations with high antimicrobial exposures tend to have high rates of AMR (10–15). In the case of syphilis, for example, macrolide resistance in *Treponema pallidum* increased to between 65 and 100% in populations exposed to more than 700 doses of macrolides/1,000 population per year but was almost universally non-existent in populations whose macrolide consumption was less than this threshold (11). Likewise populations with high consumption of cephalosporins, macrolides and fluroroquinolones have a higher prevalence of homologous AMR in circulating *N. gonorrhoeae* than lower consumption populations (15, 17). In a similar vein, *in-vitro* studies since the 1940's have demonstrated that for a range of microbes (including *N. gonorrhoeae)* resistance to a particular antimicrobial rapidly follows sustained exposure to that antimicrobial (18). An important conclusion of the studies linking AMR to antimicrobial consumption has been to make antimicrobial stewardship (limiting the usage of antimicrobials to indications where there is good evidence of net-benefit) a central pillar of the strategy to retard the emergence of AMR (16).

Because approximately 90% of gonorrhea and chlamydia infections are asymptomatic and self-limiting in MSM, roughly 90% of infections would not result in antimicrobial exposure in the absence of screening (19, 20). Thus, screening for these infections in MSM results in a substantial exposure to antimicrobials. This consideration, in conjunction with the emerging threat of untreatable STIs motivates us to interrogate what the evidence base is for recommending gonorrhea/chlamydia screening in this setting (16)? As far as we are aware, there are no randomized controlled trials that have assessed the efficacy of screening for any STI in MSM PrEP cohorts (7). Despite this, very cogent arguments could be made for screening for hepatitis C, HIV and syphilis but not *Mycoplasma genitalium* (21) in these cohorts. In this perspective piece, we explore the logical basis for making this distinction. We start with a brief review of studies that investigate the evidence for and against gonorrhea/chlamydia screening in this setting. We then propose that a Delphi consensus methodology is used to derive, assess and apply a broadly accepted set of criteria to evaluate which STIs to screen for in MSM PrEP cohorts. Finally, to illustrate the utility of the process, we derive and apply our own list of criteria as to which STIs to screen for. This process leads to the conclusion that stopping gonorrhea/chlamydia screening may offer net health benefits to MSM in PrEP cohorts.

## Evidence for and Against Screening GONORRHEA/Chlamydia

Intensive gonorrhea/chlamydia screening in PrEP cohorts has been hypothesized to provide a number of benefits including reducing the prevalence of these infections, reducing HIV transmission and engaging higher risk individuals in care (7, 22). On the other hand, intensive screening could induce AMR in these and other bacteria, it is costly and it may result in a certificate of health effect whereby those screened may feel that if they come for regular screening this gives them a bill of health that means they can relax safety devices perceived to be onerous such as condoms (2, 7, 10, 23).

None of these benefits and harms have been established empirically. In particular no randomized controlled trials have been conducted in MSM to evaluate the risks and benefits of gonorrhea/chlamydia screening (7). In heterosexuals the evidence of the efficacy of screening is mixed (24, 25). Even if the evidence in heterosexuals was strong this could not be assumed to apply to MSM PrEP cohorts due to a number of factors including a higher sexual network connectivity in this population (2). In its systematic review to inform chlamydia/gonorrhea screening guidelines, the United States Preventive Services Task Force (USPSTF) found no relevant randomized controlled trials in men and concluded: “the current evidence is insufficient to assess the balance of benefits and harms of screening for chlamydia and gonorrhea in men” (7).

Since the publication of the USPSTF's systematic review and guideline a number of relevant studies have been published. A systematic review of observational studies assessing the association between the intensity of gonorrhea/chlamydia screening and prevalence in MSM found no association between screening overall or the intensity of screening and a reduction in the prevalence of gonorrhea or chlamydia (7).

Recently published modeling studies have reached slightly different conclusions as to the efficacy of screening in MSM. One modeling study, that ignored transmission to and from the pharynx of gonorrhea and chlamydia, found that intense screening in MSM PrEP recipients in the United States, could halve the prevalence of these infections (26). A different modeling study of Belgian MSM, that included pharyngeal transmission, suggested much more moderate effects of screening (27). A key finding of both these studies was that it was the high sexual network connectivity which generated the high prevalence of gonorrhea and chlamydia.

There are wide variations in the intensity of self-reported gonorrhea/chlamydia screening in MSM between countries within Europe. An ecological study found no association between country-level screening intensity and the prevalence or incidence of these two infections (28).

Finally, a number of studies have suggested that intensive screening may be a risk factor for AMR. One analysis found that 3 ×3 screening in PrEP cohorts results in macrolide exposures that are strongly associated with the induction of AMR in a range of bacteria (10). Two ecological analyses have found positive associations between the intensity of gonorrhea/chlamydia screening and the prevalence of AMR in *N. gonorrhoeae* (29, 30).

## What Criteria Should be Used to Decide Which STIs to Screen for in MSM PrEP Cohorts?

Which criteria should be used to evaluate if screening for a particular STI in PrEP cohorts is advisable or not? A first step is evaluating if screening for the STI in question meets the modified World Health Organization criteria for screening (31). In particular, one would need to establish that the overall benefits of screening outweigh the harms (31). Whilst randomized controlled trials would be the optimal way to answer this question, to the best of our knowledge, none are planned. We do, however, have a reasonable amount of information which could be used to better guide which STIs to screen for. This includes the epidemiological evidence reviewed above as well as information pertaining to the basic biology of each STI, host immune responses and the resistogenicity of STI treatments. Ideally, a group of experts and stakeholders could be brought together and, using a Delphi-consensus-type process, develop a set of criteria to evaluate which STIs to screen for in MSM PrEP populations (32). A suitable and widely used methodology that could be used to conduct this process would be the Grading of Recommendations Assessment, Development and Evaluation (GRADE) evidence-to-decision process (33, 34). In such a process the experts would first select a set of criteria to use to evaluate which STIs to screen for in MSM PrEP cohorts. In the second phase, they would review the relevant literature to decide to what extent the evidence supports screening per criterion and STI (Table 1). In each phase the experts would provide their initial answers which are then summarized, anonymized and shared with the group by a facilitator. The experts are then invited to revise their answers and the process repeated until either consensus is reached or a pre-specified outcome is attained (32, 35). A similar process was successfully used to derive the widely used new clinical criteria for defining septic shock (32).

**Table 1:** Non-exclusive list of possible criteria for evaluating net utility of screening six specific STIs in MSM PrEP cohorts^#^.

	**Ng**	**Mg**	**Ct**	**Tp**	**HCV**	**HIV**
**STEP 1: ASSESS IF HOST-PATHOGEN INTERACTIONS ARE AMENABLE TO SCREENING**						
1. Undetected infection typically associated with serious adverse clinical outcomes	1	1	1	4	4	5
2. Long period between infection and disease onset	1	1	1	3	5	5
3. Not spontaneously cleared by immune system	0	0	0	3	3	5
4. No immunity from naturally cleared infection	3	3	2	2	5	5
Total	5	5	2	12	17	20
**STEP 2: ASSESS THE RISK OF INDUCING AMR**						
1. Low risk of inducing AMR in pathogen itself given standard therapy	0	0	4	4	5	5
2. Low risk of inducing AMR in microbiome given standard therapy	0	1	2	4	5	5
Total	0	1	6	8	10	10

*Each STI is scored for each criterion on a scale from 0 (highly unlikely) to 5 (very likely) according to probability that screening would result in a positive outcome according to this criterion^#^*.

# *This scoring is based on a subjective assessment of the author's evaluation of the scientific literature. This assessment was performed in the absence of systematic reviews on each of these criteria/pathogen combinations*.

*Ng, Neisseria gonorrhoeae; Mg, Mycoplasma genitalium; Ct, Chlamydia trachomatis; Tp, Treponema pallidum; HCV, hepatitis C; HIV, Human Immunodeficiency Virus*.

## An Illustration of Possible STI-screening-criteria

Whilst this Delphi-process is considerably beyond the scope of this opinion piece, we illustrate how the criteria selection phase may unfold by outlining the criteria which we would select. We would choose a two-step, 6-criteria process for evaluating which STIs to screen for (Table 1). Based on our reading of the literature, we then provide a score for each of these 6 criteria relating to each of 6 STIs—chlamydia, HIV, hepatitis C, gonorrhea, *M. genitalium*, and syphilis. Each is scored from 0 to 5 according to whether they are highly unlikely (scored 0) to very likely (scored 5) to result in a net utility for screening (Table 1).

### Step 1: Assess if the Biology of STI-host Interactions Make the STI Amenable to Screening

Numerous aspects of the way *N. gonorrhoeae* circulates in MSM decreases the probability that screening will be beneficial. Symptomatic disease is thought to typically occur soon (2–21 days) after infection. If symptoms do not develop, the infection (particularly in the pharynx and rectum) tends to persist in a low abundance state for up to 6 months (20). Highly exposed individuals develop a type-specific immunity but this immunity is largely ineffective in low exposure individuals (20, 36). The vast majority of *N. gonorrhoeae* infections are asymptomatic and self-limiting in this population (19, 20). Screening is far more likely to diagnose infections in the 6 month asymptomatic tail phase (when *N. gonorrhoeae* abundance is likely lower and therefore less infectious) than in the acute first weeks post infection. These features reduce the probability that screening will decrease either symptomatic infections or *N. gonorrhoeae* transmissions—assuming that the low abundance infections are less infectious. Similar considerations apply to *C. trachomatis* and *M. genitalium*. In the case of *C. trachomatis* there is however better evidence that treatment of *C. trachomatis* results in “arrested immunity” and thereby paradoxically increases the probability of reinfection and may even lead to increases in prevalence (37, 38). As a result, all 3 of these STIs score poorly for being amenable to screening when assessed by these amenability criteria (Table 1).

The assessments for syphilis, hepatitis C and HIV are, however, very different. Each has a relatively long latent period, each results in serious disease and the probability of spontaneous clearance ranges from moderate (syphilis) to close to zero (HIV) (39–41). As a result, screening is likely to both reduce the probability of serious disease in infected individuals and the probability of onward transmission. They thus have high screening-utility-scores for the first step (Table 1).

### Step 2: Assess the Risk of Inducing AMR

**Figure 1:**
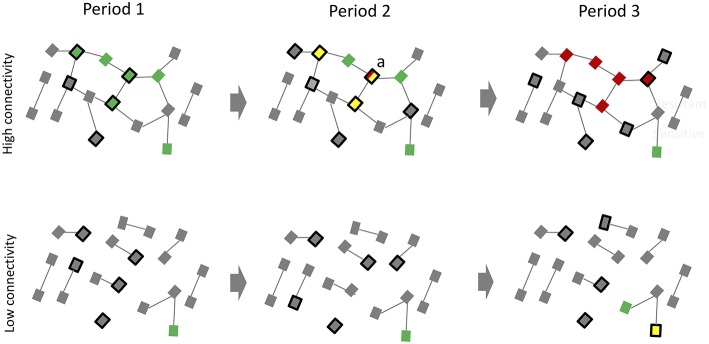
An illustration of how frequent screening for *N. gonorrhoeae* in MSM preexposure prophylaxis (PrEP) populations may have little effect on reducing prevalence of *N. gonorrhoeae* but result in the development of antimicrobial resistance. Period (1) The high sexual network connectivity of a typical PrEP cohort (top) translates into a high equilibrium prevalence of *N. gonorrhoeae* (green squares). Period (2) Active screening of a quarter of this population (black bordered squares) results in a lower *N. gonorrhoeae* prevalence in period 2 but at the expense of an altered resistome (yellow squares represent individuals with *N. gonorrhoeae* cleared via antibiotics in preceding period). Because the network connectivity remains unchanged, *N. gonorrhoeae* tends to return to its equilibrium prevalence. This places recently cured individuals (such as individual “a”) at high risk of reinfection at a time when their resistomes are enriched with resistance genes. An early *N. gonorrhoeae* reinfection in “a” is able to take up these resistance genes via transformation and become resistant to the antibiotics used to treat *N. gonorrhoeae* (red squares). Period (3) If there is ongoing high exposure to antibiotics these less susceptible *N. gonorrhoeae* strains will have a fitness advantage over more susceptible strains. These dynamics would be predicted to favor the emergence and spread of resistant *N. gonorrhoeae*. By period 3, *N. gonorrhoeae* has returned to its equilibrium prevalence for this degree of network connectivity but now most strains are resistant. The degree of connectivity in the low connectivity population (bottom) is so low that *N. gonorrhoeae* remains at a very low prevalence. Even extensive screening is unlikely to result in sufficient antibiotic exposure to provide *N. gonorrhoeae* access to resistance genes or a fitness advantage for resistance strains (Uninfected individuals: gray squares; Edges between squares represent sexual relationships).

As noted above, gonorrhea/chlamydia screening in MSM can result in exposure levels to macrolides/cephalosporins that are strongly associated with resistance in *N. gonorrhoeae, Treponema pallidum* and a range of other bacteria. Macrolides are typically recommended as first line therapy for *M. genitalium*, but involve a 10% risk of inducing macrolide AMR in *M. genitalium* per treatment (42). These considerations suggest that screening for *N. gonorrhoeae* and *M. genitalium* run a high risk of inducing AMR in both the bacteria themselves and the resident microbiomes. A single dose of azithromycin, for example, has been shown to have adverse effects on the recipient's microbiome and resistome (including macrolide resistance mechanisms) that persists for up to 6 months in the oropharynx and 48 months in the colon (43, 44). If an azithromycin recipient acquires a new *N. gonorrhoeae* infection in this time period, it could acquire the macrolide resistance mutations from the commensal population (via horizontal gene transfer) and thereby become less-susceptible to macrolides (Figure 1) (2, 45). Various studies have provided suggestive evidence that *N. gonorrhoeae* has acquired penicillin, cephalosporin, and macrolide resistance via this type of mechanism (45, 46). As a result, *N. gonorrhoeae* and *M. genitalium* once again score poorly in the second step criteria (Table 1).

Screening for hepatitis C and HIV, on the other hand, involves little risk of these pathogens and commensals acquiring resistance if standard treatment protocols are followed. Likewise, if penicillin is used for therapy, screening for syphilis involves an extremely low risk of inducing AMR in *T. pallidum* and a lower risk of inducing wide-ranging resistance in the microbiome than other antimicrobial classes such as the macrolides and extended spectrum cephalosporins (39, 47). As a result, these three STIs have high scores for screening in both steps one and two.

If these two steps are followed, then a strong case can be made that screening for *T. pallidum*, hepatitis C and HIV is likely to reduce disease in individuals screened, reduce transmission and involve little or no risk of inducing AMR. Conversely, screening for *N. gonorrhoeae, C. trachomatis* and *M. genitalium* is less likely to result in decreasing disease in those screened, less likely to reduce prevalence and particularly for *N. gonorrhoeae* and *M. genitalium*, more likely to induce AMR. Modeling studies that include the effect of screening on (1) prevalence/incidence of each STI, (2) the probability of AMR emergence and (3) cost-effectiveness analyses would be useful to provide further evidence as to the net utility of screening. The use of antimicrobials has also been associated with the genesis of AMR in pathobionts and commensals not targeted by the antimicrobials (bystander selection) (48). Antimicrobials also have a range of other deleterious effects such as on the health of the microbiome which may be long lasting. Optimally studies evaluating the net utility of screening should include these as secondary outcomes.

## Conclusion

PrEP populations typically have high rates of partner change, partner concurrency and a low prevalence of condom use (1, 5). These behaviors generate dense sexual networks which result in high equilibrium prevalences of most STIs [reviewed in (2), (49); Figure 1]. The prevalence of *N. gonorrhoeae, C. trachomatis* and *M. genitalium*, for example, are each typically 8–17% in PrEP cohorts (7, 50). The sexual networks in these cohorts are so dense that even 3 ×3 screening/treatment for *N. gonorrhoeae, C. trachomatis*, and *M. genitalium* has not been found to result in a decrease in the prevalence of these infections (5, 7, 9, 27, 50). Screening does however result in considerable increases in antimicrobial exposure with the attending risk of AMR (10). These considerations suggest the need to revisit the evidence base for screening for these infections. The urgency for this stems from predictions such as those from the O'Niell report that antimicrobial resistant infections will cause more deaths worldwide than cancer in 2050 (16). This is not inevitable. We know that the predominant determinant of the AMR epidemic is excess use of antimicrobials (14, 16). Populations with low consumption of antimicrobials have corresponding low prevalences of AMR (12–15). If providers and MSM PrEP clients consider the risks and benefits of screening for gonorrhea/chlamydia via a Delphi-consensus process they may jointly decide to continue screening for HIV, hepatitis C and syphilis but reduce or stop screening for gonorrhea/chlamydia. One option would be to phase out gonorrhea/chlamydia screening in a controlled and staggered way as part of a pragmatic study design. This would provide further evidence on the net benefits and risks of screening.

## Data Availability

No datasets were generated or analyzed for this study.

## Author Contributions

The author confirms being the sole contributor of this work and has approved it for publication.

### Conflict of Interest Statement

The author declares that the research was conducted in the absence of any commercial or financial relationships that could be construed as a potential conflict of interest.
